# Perioperative changes in the microbiome during rectal cancer surgery: exploratory analysis of the National Institute for Health and Care Research (NIHR) IntAct trial

**DOI:** 10.1093/bjs/znaf199

**Published:** 2025-09-30

**Authors:** Jack A Helliwell, Caroline H Chilton, Caroline Young, Emma V Clark, Lyndsay Wilkinson, Alba Fuentes Balaguer, Daniel Bottomley, Julie Croft, Neil Corrigan, Andrew Kirby, Philip Quirke, Deborah D Stocken, David G Jayne, Henry M Wood

**Affiliations:** Leeds Institute of Medical Research, University of Leeds, Leeds, UK; Leeds Institute of Medical Research, University of Leeds, Leeds, UK; Department of Histopathology, Leeds Teaching Hospitals NHS Trust, Leeds, UK; Leeds Institute of Medical Research, University of Leeds, Leeds, UK; Leeds Institute of Medical Research, University of Leeds, Leeds, UK; Leeds Institute of Medical Research, University of Leeds, Leeds, UK; Leeds Institute of Medical Research, University of Leeds, Leeds, UK; Leeds Institute of Clinical Trials Research, University of Leeds, Leeds, UK; Leeds Institute of Clinical Trials Research, University of Leeds, Leeds, UK; Leeds Institute of Medical Research, University of Leeds, Leeds, UK; Leeds Institute of Medical Research, University of Leeds, Leeds, UK; Leeds Institute of Clinical Trials Research, University of Leeds, Leeds, UK; Leeds Institute of Medical Research, University of Leeds, Leeds, UK; Leeds Institute of Medical Research, University of Leeds, Leeds, UK

## Abstract

**Background:**

The gut microbiome may influence postoperative outcomes after rectal cancer surgery, including anastomotic leak. However, perioperative microbiome dynamics and their association with outcomes remain poorly understood. The aim of this study was to characterize changes in the rectal microbiome in patients undergoing rectal cancer surgery within the National Institute for Health and Care Research (NIHR) IntAct trial.

**Methods:**

Rectal swabs were collected at baseline, day of surgery, and postoperative day 3–5. DNA was extracted for 16S ribosomal RNA (rRNA) sequencing and collagenase-producing organisms were identified by culture. Associations between microbiome composition and clinical variables were analysed.

**Results:**

A total of 202 patients were included (mean age 65 years; 69.8% male). At baseline, smoking status explained 3.2% of variation in beta-diversity (*P* = 0.046). On the day of surgery, beta-diversity was associated with hospital site (11.1%; *P* = 0.033), mechanical bowel preparation (2.6%; *P* = 0.024), and preoperative oral antibiotics (1.0%; *P* = 0.020). After surgery, hospital site (16.3%; *P* < 0.001), a defunctioning stoma (2.9%; *P* = 0.003), and preoperative oral antibiotics (1.6%; *P* = 0.006) influenced beta-diversity. Alpha-diversity decreased over time, with postoperative increases in *Enterococcus* and *Prevotella*. A defunctioning stoma was associated with lower alpha-diversity and increased *Pseudomonas* and *Streptococcus*. No significant difference in alpha- or beta-diversity was observed between patients with and without anastomotic leak, although subtle differences in taxa of low abundance were detected and 43.6% of postoperative samples demonstrated collagenase activity.

**Conclusion:**

This is the largest study to date describing perioperative microbiome changes in patients undergoing rectal cancer surgery. Measurable shifts in the microbiome were observed, with small differences between patients with and without anastomotic leak. Further research is needed to explore the clinical significance of these microbiome changes.

## Teaser

This analysis explored perioperative microbiome changes in patients undergoing rectal cancer surgery within the National Institute for Health and Care Research (NIHR) IntAct trial. Alpha-diversity decreased during treatment, with postoperative increases in *Enterococcus* and *Prevotella*. While small microbial differences were observed in patients with anastomotic leak, their clinical significance remains unclear and warrants further investigation.

## Introduction

The importance of the gut microbiome is increasingly recognized, with its influence extending across a range of clinical conditions^1^. In rectal cancer surgery, its role in the development of postoperative complications has emerged as an area of growing interest^2^. Among these complications, anastomotic leak remains one of the most feared, due to its association with increased morbidity, mortality, and healthcare costs^3,4^. Despite efforts to reduce known risk factors—such as ensuring a tension-free anastomosis, confirming adequate blood supply, and optimizing surgical technique—the incidence of anastomotic leak remains stubbornly persistent, with reported rates of 10–15% after rectal cancer surgery^5,6^.

Preclinical studies suggest that the gut microbiome can impair anastomotic wound healing^7^. Factors such as surgical injury, ischaemia, and malnutrition appear to disrupt microbial populations, increasing pathogenic bacteria, while reducing protective strains^8^. This dysbiosis may promote the colonization of the anastomotic site by pathogenic bacteria, such as *Enterococcus faecalis* and *Pseudomonas aeruginosa*. These organisms produce collagenolytic enzymes and activate host matrix metalloproteinases, leading to collagen degradation *in vitro* and anastomotic leak in animal models^7^. While these preclinical studies provide valuable insights, data from human studies remain limited. Few studies have tracked changes in the microbiome over multiple time points in colorectal surgery and those that have often lack sufficient patient numbers to investigate potential associations with clinical outcomes, such as anastomotic leak^9^.

The aim of this exploratory analysis, conducted as a substudy of the National Institute for Health and Care Research (NIHR) IntAct trial—a pan-European multicentre RCT—was to address this gap^10^. Specifically, it sought to: describe perioperative changes in the microbiome of rectal cancer surgery patients across defined time points, highlighting overall trends within the study population; analyse the microbiome of rectal cancer patients, with an emphasis on key explanatory variables, including patient characteristics, disease factors, and treatment approaches; and explore the microbiome profile of rectal cancer patients, with a specific focus on anastomotic leak as a key outcome variable.

## Methods

### Study design

A microbiome substudy was conducted across UK centres participating in the NIHR IntAct trial. IntAct was a pan-European multicentre RCT that recruited 768 patients across 28 centres in eight countries. It compared surgery with intraoperative fluorescence angiography (IFA) with standard care (surgery without IFA) to assess the impact on anastomotic leak rates after anterior resection for rectal cancer^10,11^. The microbiome substudy was limited to UK centres due to logistical constraints—specifically, the need for timely analysis of rectal swabs. Ethical approval was obtained from the Research Ethics Committee (REC) (17/NW/0193) and Health Research Authority (HRA). The trial was prospectively registered with the ISRCTN Registry (ISRCTN13334746).

### Sample collection

Rectal swabs (Sigma-Transwabs and M40 charcoal swabs) were collected at three time points: baseline (before surgery and bowel preparation); day of surgery; and postoperative day 3–5. For patients performing bowel preparation at home, the baseline sample was obtained during a preoperative hospital visit. At each time point, a member of the research team collected samples before transferring them for analysis. Samples were analysed centrally using two approaches: 16S ribosomal RNA (rRNA) sequencing; and microbial culture to isolate and quantify bacterial collagenase production.

### 16S rRNA sequencing

DNA was extracted from rectal swabs using the QIAmp DNA Stool Mini Kit^12^. Libraries were prepared following Earth Microbiome Project protocols to amplify the V4 region of the 16S ribosomal gene^13^. Sequencing was performed on Illumina HiSeq3000 and NextSeq2000 (2 × 150 bp reads). Adapters were trimmed with Cutadapt and processed in QIIME2^14,15^. Denoising and merging were done using DADA2^16^. Taxonomy was assigned using QIIME2’s BLAST classifier aligned to the SILVA database version 132^17–19^.

Alpha-diversity was measured using the Shannon index, which captures richness and evenness of taxa within samples. Beta-diversity was calculated using Bray–Curtis distances, which are a measure of compositional differences between samples^20^. Associations between beta-diversity and clinical metadata were assessed using permutational multivariate analysis of variance (PERMANOVA) with the adonis2 function in R. To identify associations between specific taxa and clinical variables, version 2 of Multivariate Associations with Linear Models (MaAsLin2) was used; it performs multivariable linear modelling with false-discovery-rate correction to account for multiple testing^21^.

### Identification of collagenolytic bacteria and quantification of collagenase production

Collagenolytic bacteria were isolated from rectal swabs using a skimmed milk method as previously described^22^. Samples were plated on aerobic and anaerobic media containing skimmed milk and incubated at 37°C. Colonies showing zones of hydrolysis were purified and identified using matrix-assisted laser desorption/ionization time-of-flight (MALDI-TOF) mass spectrometry.

Collagenase activity for all identified collagenolytic isolates was quantified against type I and type IV collagen using the EnzChek™ Gelatinase/Collagenase Assay Kit. Isolates were subcultured and grown overnight in tryptic soy broth (TSB), then diluted 1 : 10 in fresh TSB and inoculated into 96-well plates with 25 μg/ml collagen substrate and reaction buffer. Florescence (495 nm/515 nm) was measured over time and quantified against a *Clostridium* collagenase standard curve (0.06–1 U/ml). Absorbance at 595 nm was measured to correct for bacterial load.

### Clinical data

Clinical metadata were collected for all patients and analysed alongside sequencing and collagenase data. Variables included: sample time point, hospital site, age, sex, ethnicity, smoking status, tumour stage and position, neoadjuvant therapy, mechanical bowel preparation type, preoperative oral antibiotics, anastomosis level, defunctioning stoma, circumferential resection margin involvement, indocyanine green administration, and anastomotic leak grade (per the International Study Group definition)^23^.

## Results

### Study population

A total of 202 patients were recruited to the substudy and provided usable microbiome samples (*Table 1*). The majority were male (141 patients (69.8%)), with a mean age of 65 (range 30–89) years. Most did not receive neoadjuvant therapy (138 patients (68.3%)). All but one patient underwent mechanical bowel preparation (201 patients (99.5%)), with oral mechanical bowel preparation being most common (151 patients (74.8%)). Seventeen patients (8.4%) also received preoperative oral antibiotics. A defunctioning stoma was performed in the majority of patients (143 patients (70.8%)). The overall anastomotic leak rate (grades A, B, and C) was 19.8% (40 patients).

**Table 1:** Demographic and clinical characteristics of patients in the microbiome substudy (*n* = 202)

Category	Values
Sex	Male: 141 (69.8) Female: 61 (30.2)
Age (years)	Mean: 65 (range 30–89)
Ethnicity	White: 192 (95.0) Asian: 6 (3.0) Black (Caribbean): 2 (1.0) NA: 2 (1.0)
Smoking status	Current smoker: 15 (7.4) Never smoked: 100 (49.5) Ex-smoker: 71 (35.1) NA: 16 (7.9)
Tumour position	Above peritoneal reflection: 70 (34.7) At peritoneal reflection: 68 (33.7) Below peritoneal reflection: 62 (30.7)
Tumour T category	TX: 2 (1) T1: 6 (3) T2: 48 (23.8) T3: 134 (66.3) T4a: 5 (2.5) NA: 7 (3.5)
Neoadjuvant therapy	Yes: 60 (29.7) No: 138 (68.3) NA: 4 (2)
Mechanical bowel preparation	Enema only: 12 (5.9) Mechanical (oral) only: 151 (74.8) Mechanical (oral) plus enema: 35 (17.3) None: 1 (0.5) NA: 3 (1.5)
Preoperative oral antibiotics	Yes: 17 (8.4) No: 181 (89.6) NA: 4 (2)
ASA grade	Grade I: 42 (20.8) Grade II: 136 (67.3) Grade III: 22 (10.9) NA: 2 (1)
Type of resection	High anterior resection: 33 (16.3) Low anterior resection: 151 (74.8) NA: 18 (8.9)
ICG administration	Yes: 104 (51.5) No: 98 (48.5)
CRM involvement	Yes: 40 (19.8) No: 152 (75.2) NA: 8 (4)
Defunctioning stoma	Yes: 143 (70.8) No: 44 (21.8) NA: 15 (7.4)
Anastomotic leak (grades A, B, and C)^23^	Yes: 40 (19.8) No: 162 (80.2)

Values are *n* (%) unless otherwise indicated. NA, data not available; ICG, indocyanine green; CRM, circumferential resection margin.

### Sample collection

All 202 patients (100%) were included in the 16S rRNA sequencing analysis, with samples collected at the following time points: 98 (49%) at baseline, 180 (89%) intraoperatively, and 103 (51%) after surgery. In addition, 198 patients (98%) were included in the culture-based microbiome analysis, with samples received at the following time points: 101 (50%) at baseline, 198 (98%) intraoperatively, and 110 (54%) after surgery.

### Sequencing metrics

Each sample produced between 1862 and 1 539 340 denoised sequences (median 55 727, mean 83 684). Genera commonly associated with colorectal cancer and healthy stool, including *Bacteroides*, *Prevotella*, and *Faecalibacterium*, were prevalent^12,24^.

### Characteristics of cohort

**Fig. 1:**
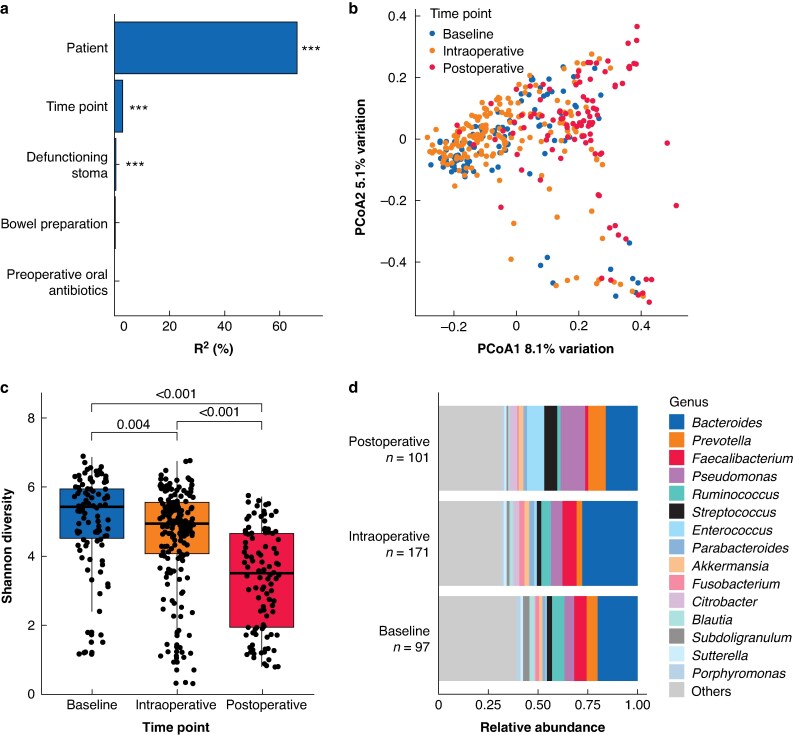
Microbial profiling of the study cohort **a** PERMANOVA (adonis2) analysis of Bray–Curtis beta-diversity across all metadata categories. R^2^ values represent the proportion of variation explained. ***P < 0.001. **b** PCoA plot of Bray–Curtis distances, with samples coloured by time point. **c** Shannon alpha-diversity by time point. Mann–Whitney *U* test *P* values are given. **d** Cumulative relative abundance of bacterial taxa across all samples, grouped by time point. PERMANOVA, permutational multivariate analysis of variance; PCoA, principle coordinate analysis.

Initial inspection of the cohort (*Fig. 1a*) revealed that the individual patient was associated with the largest proportion of variation, explaining 66% of beta-diversity (*P* < 0.001). Time point of sample collection accounted for 3.1% of the variance and the presence of a defunctioning stoma accounted for 0.5% (both *P* < 0.001). Type of mechanical bowel preparation and preoperative oral antibiotics were associated with non-significant amounts of variation. Together, these five factors accounted for all clinical variation between samples, making further testing for associations with additional variables redundant.

### Perioperative changes in the microbiome across time points

Principle coordinate analysis (PCoA) of beta-diversity revealed that baseline and intraoperative samples clustered together, while postoperative samples formed a distinct cluster (*Fig. 1b*). Alpha-diversity decreased between the baseline and intraoperative time points and again between the intraoperative and postoperative time points (*Fig. 1c*).

Postoperative samples showed increases in *Enterococcus* and *Prevotella*, and relative decreases in *Faecalibacterium* and *Ruminococcus* (*Fig. 1d*). Additionally, there was a visible increase in *Pseudomonas* and a decrease in *Bacteroides* between intraoperative and postoperative time points. However, these changes were largely influenced by the presence of a defunctioning stoma and thus neither of these shifts was significant after multivariate analysis (*Table S1)*.

No consistent changes in common taxa were observed between the baseline and intraoperative time points (*Fig. 1d*).

### Time point-specific analysis of the microbiome

At the baseline time point (*Table 2*), smoking status accounted for 3.2% of the variation in beta-diversity (*P* = 0.046), but no individual taxa were associated (*Table S1*). Notably, no association was observed between beta-diversity and neoadjuvant therapy.

**Table 2:** Associations between clinical metadata and microbiome composition at baseline, intraoperative, and postoperative time points (PERMANOVA (adonis2) analysis of Bray–Curtis distances)

Category	Proportion of variation (R^2^)	*P*
**Baseline samples**		
Hospital site	0.156	0.125
Anastomotic leak	0.111	0.534
Neoadjuvant therapy	0.009	0.824
Tumour position	0.028	0.130
T category	0.034	0.655
CRM involvement	0.014	0.153
Ethnicity	0.037	0.348
Sex	0.012	0.406
Smoking	0.032	0.046*
Age	0.010	0.727
**Intraoperative samples**		
Hospital site	0.111	0.033*
Anastomotic leak	0.007	0.382
Preoperative oral antibiotics	0.010	0.020*
Neoadjuvant therapy	0.007	0.437
Mechanical bowel preparation	0.026	0.024*
Tumour position	0.011	0.802
T category	0.025	0.624
CRM involvement	0.008	0.146
Ethnicity	0.036	0.183
Sex	0.008	0.101
Smoking	0.092	0.992
Age	0.006	0.553
**Postoperative samples**		
Hospital site	0.163	<0.001*
Anastomotic leak	0.012	0.415
Preoperative oral antibiotics	0.016	0.006*
Neoadjuvant therapy	0.008	0.911
Mechanical bowel preparation	0.044	0.058
Defunctioning stoma	0.029	0.003*
Tumour position	0.024	0.460
T category	0.041	0.892
CRM involvement	0.013	0.350
Ethnicity	0.013	0.360
Sex	0.013	0.309
Smoking	0.022	0.559
Age	0.014	0.221
ICG administration	0.018	0.087

^*^Statistically significant. PERMANOVA, permutational multivariate analysis of variance; CRM, circumferential resection margin; ICG, indocyanine green.

Among intraoperative samples (*Table 2*), the largest proportion of beta-diversity was explained by hospital site (11.1% of variation; *P* = 0.033), with smaller proportions linked to type of mechanical bowel preparation (2.6% of variation; *P* = 0.024) and preoperative oral antibiotics (1% of variation; *P* = 0.020). Patients who underwent rectal enema bowel preparation only had higher levels of *Ruminococcus* compared with those who underwent oral mechanical bowel preparation alone. The use of preoperative oral antibiotics was associated with relative increases in *Ruminococcus*, *Eubacterium*, Lachnospiraceae, and *Bifidobacterium* (*Table S1*).

Among postoperative samples (*Table 2*), beta-diversity was associated with the hospital site (16.3% of variation; *P* < 0.001), the presence of a defunctioning stoma (2.9% of variation; *P* = 0.003), and the use of preoperative oral antibiotics (1.6% of variation; *P* = 0.006).

### Impact of a defunctioning stoma on the microbiome

**Fig. 2:**
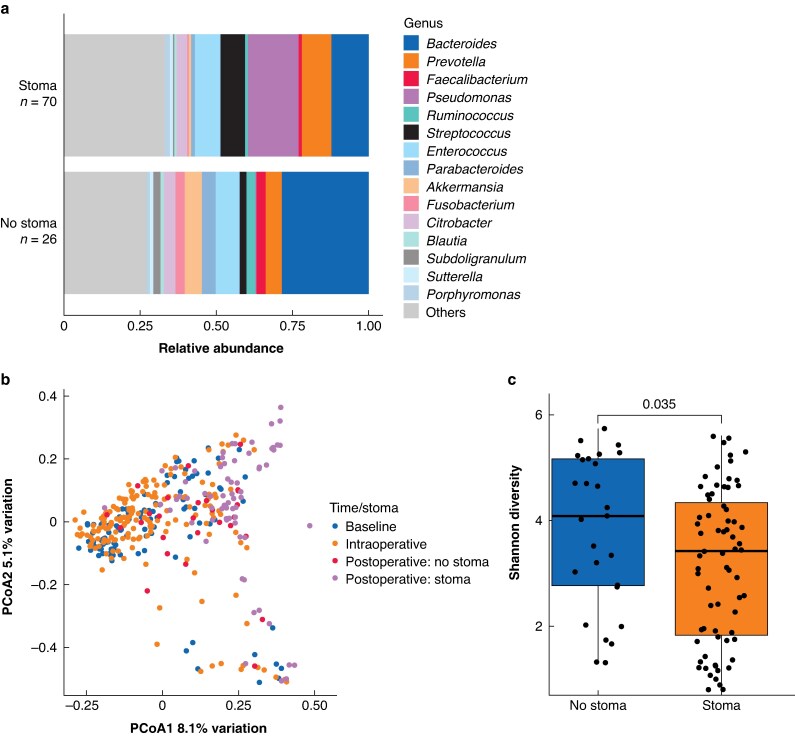
Impact of a defunctioning stoma on the postoperative microbiome **a** Cumulative relative abundance of the most abundant bacterial taxa, stratified by the presence or absence of a defunctioning stoma. **b** PCoA plot of Bray–Curtis beta-diversity, grouped by time point and stoma status. **c** Shannon alpha-diversity of postoperative samples, stratified by stoma status. PCoA, principle coordinate analysis.

Postoperative samples from patients with a defunctioning stoma showed distinct clustering in PCoA of beta-diversity, separate from both baseline and intraoperative samples (*Fig. 2b*). In contrast, samples from patients without a defunctioning stoma clustered more closely with earlier time points. Patients with a defunctioning stoma showed a significant reduction in alpha-diversity (*Fig. 2c*) and notable increases in *Pseudomonas* and *Streptococcus*, with decreases in *Bacteroides*, *Akkermansia*, and *Parabacteroides* (*Fig. 2a* and *Table S1*).

To investigate whether these differences were attributable to variations in anastomotic height, anastomotic height was included in PERMANOVA analysis. This revealed no significant association between anastomotic height and beta-diversity (*P* = 0.380). Missing data for anastomotic height precluded its inclusion in the original model.

**Fig. 3:**
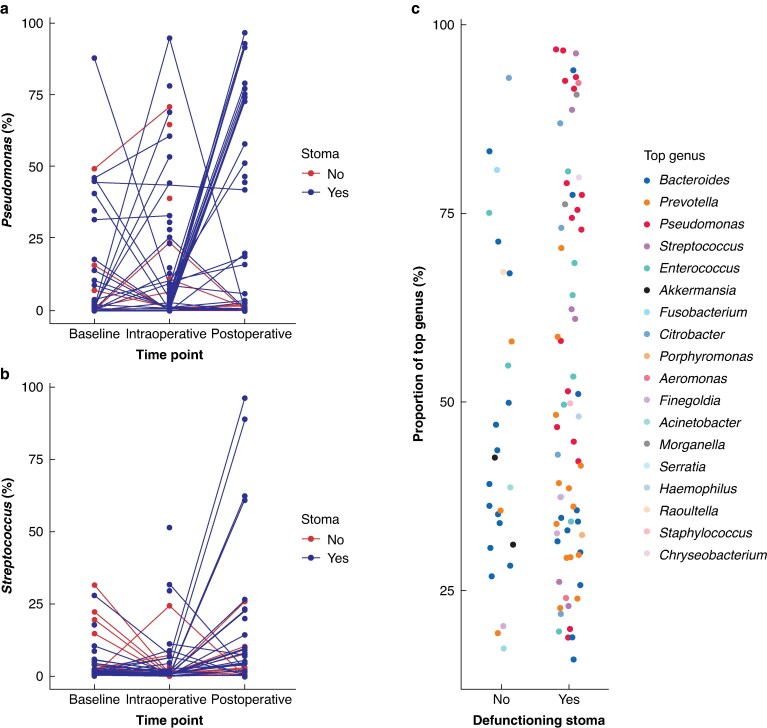
Postoperative spikes in specific taxa associated with a defunctioning stoma **a** Relative abundance of *Pseudomonas* across time points, stratified by stoma status. **b** Relative abundance of *Streptococcus* across time points, stratified by stoma status. **c** Proportion of most abundant genus in each postoperative sample, stratified by presence or absence of a defunctioning stoma.

Given the distinct microbiome profile and reduced alpha-diversity observed in postoperative samples from patients with a defunctioning stoma, the authors further explored changes in key taxa at the individual patient level. The authors initially focused on the two taxa that showed the most prominent differences, *Pseudomonas* and *Streptococcus*, tracking their changes across time points within individual patients (*Fig. 3a,b*). In a subset of patients, marked increases (>50% of the microbiome) in *Pseudomonas* (13 of 77 (17%)) and *Streptococcus* (4 of 77 (5%)) were observed after surgery. These spikes occurred exclusively in patients with a defunctioning stoma. In the case of a *Pseudomonas* spike, this often accounted for >70% of the microbiome signal (*Fig. 3a*).

To determine whether other taxa became highly prevalent after surgery and whether this was associated with the presence of a defunctioning stoma, the authors plotted the most common taxa in each postoperative sample (*Fig. 3c*). In patients without a defunctioning stoma and in patients with a defunctioning stoma, 9 of 26 (34.6%) and 31 of 70 (44.3%) respectively had a single genus that accounted for >50% of the microbiome signal. The dominant taxa in these groups differed. *Enterococcus* and *Prevotella* were more common in samples from patients without a defunctioning stoma. These taxa were also seen in samples from patients with a defunctioning stoma, but *Pseudomonas*, *Streptococcus*, *Morganella*, *Aeromonas*, and *Chryseobacterium* were more prevalent.

### Anastomotic leak and the microbiome

**Fig. 4:**
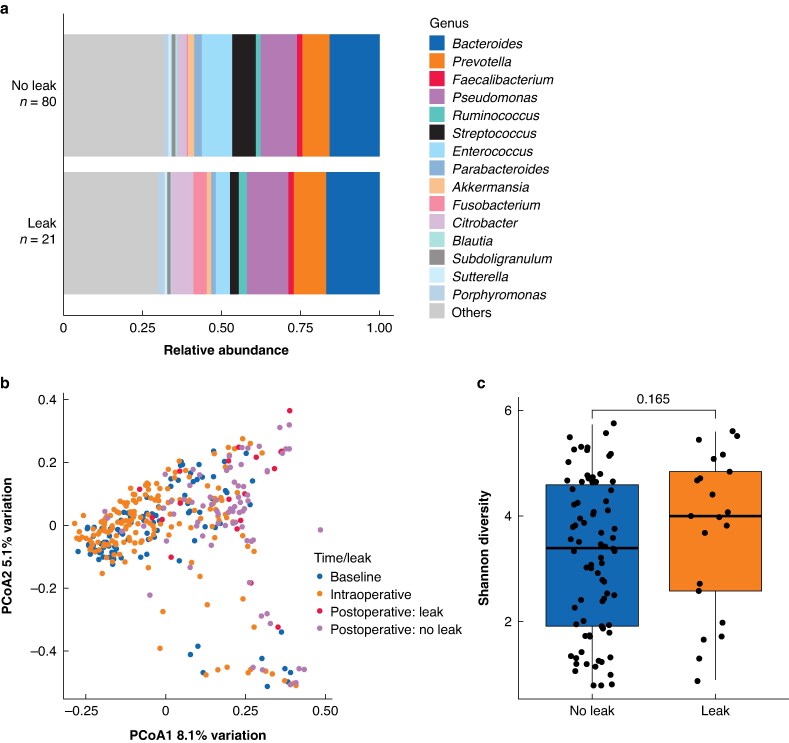
Comparison of the postoperative microbiome by anastomotic leak status **a** Cumulative relative abundance of bacterial taxa in postoperative samples, stratified by presence or absence of anastomotic leak. **b** PCoA plot of Bray–Curtis beta-diversity, grouped by time point and anastomotic leak status. **c** Shannon alpha-diversity of postoperative samples, stratfied by anastomotic leak status. PCoA, principle coordinate analysis.

Focusing on taxa implicated in anastomotic leak in preclinical studies, such as *Enterococcus* and *Pseudomonas*, their proportions in patients with and without anastomotic leak were examined. *Pseudomonas* was slightly increased in patients with anastomotic leak, while *Enterococcus* was slightly decreased (*Fig. 4a*). Neither change was significant and both were dwarfed by changes associated with a defunctioning stoma.

Consistent with PERMANOVA analysis, visual inspection of beta-diversity PCoA plots showed no distinct clustering of postoperative samples based on anastomotic leak status (*Fig. 4b*).

Although anastomotic leak did not significantly affect overall beta-diversity, it was associated with a higher relative abundance of *Hungatella* and *Eisenbergiella*, and a reduced abundance of *Barnesiella* on the day of surgery (*Table S1*); however, all were present at low levels (median <1% per sample). After surgery, anastomotic leak was also linked to an increased relative abundance of *Eubacterium*, although this was <0.1% of the signal for most samples (*Table S1*).

No significant difference in alpha-diversity was observed between patients with and without anastomotic leak (*Fig. 4c*).

### Microbiological culture-based analysis of collagenase-producing bacteria

To supplement the sequencing data, culture-based methods were used to isolate collagenase-producing organisms and quantify their collagenase activity. A total of 409 samples from 198 patients were analysed for collagenase activity. Of these, 152 samples (37.2%) demonstrated evidence of collagenase activity. The proportion of samples with collagenase activity at each time point was as follows: 37 of 101 (36.6%) at baseline, 67 of 198 (33.8%) intraoperatively, and 48 of 110 (43.6%) after surgery. The most commonly identified bacterial species with collagenase activity were: *P. aeruginosa* (62 isolates (40.8%)), *E. faecalis* (33 isolates (21.7%)), and *Proteus mirabilis* (18 isolates (11.8%)) (*Table 3*).

**Table 3:** Concordance between culture of collagenase-producing organisms and 16S rRNA sequencing analysis

Organism of interest, genus	Culture, cultured species (no. of isolates)	Higher proportion of 16S sequencing reads mapping to organism of interest	
		Where collagenase-producing organism of interest cultured	Where any collagenase-producing species cultured
*Pseudomonas*	*Pseudomonas aeruginosa* (62), *Pseudomonas otitidis* (1)	<0.001	<0.001
*Enterococcus*	*Enterococcus faecalis* (33)	<0.001	0.016
*Proteus*	*Proteus mirabilis* (18)	<0.001	0.018
*Staphylococcus*	*Staphylococcus epidermis* (11)	<0.001	0.627
*Clostridium senso stricto 1*	Clostridium perfringens* (8)	0.003	0.913
*Aeromonas*	*Aeromonas hydrophila* (6), *Aeromonas veronii* (3)	<0.001	0.020
*Prevotella*	Prevotella bivia* (5), Prevotella disiens* (3), Prevotella buccalis* (1), Prevotella timonensis* (1)	<0.001	0.635
*Serratia*	*Serratia marcescens* (2)	<0.001	0.688
*Porphyromonas*	Porphyromonas somerae* (1)	Not enough samples	0.151
*Arthrobacter*	*Arthrobacter cumminsii* (1)	Not present	Not present
*Bacillus*	*Bacillus amyloliquefaciens* (1), *Bacillus cereus* (1), *Bacillus pumilus* (1), *Bacillus simplex* (1), *Bacillus weihenstephanensis* (1)	0.605	0.279
*Paeniclostridium*	Paeniclostridium sordellii* (1)	0.544	0.366
*Kocuria*	*Kocuria rhizophilia* (1)	<0.001	0.025
*Stenotrophomonas*	*Stenotrophomonas maltophilia* (1)	Not enough samples	0.703
Unknown ID	Unknown ID (2)	NA	NA

For every putative collagenolytic genus, the names and numbers of isolates of each species cultured are given. Mann–Whitney *U* test *P* values of the difference in proportions of each genus in samples with *versus* without collagenase activity are given, as well as Mann–Whitney *U* test *P* values of the difference in proportions of each genus in samples where species from that genus were cultured *versus* not cultured. *Obligate anaerobes. rRNA, ribosomal RNA; ID, identifier.

After isolation of collagenolytic bacteria, collagenase activity was quantified against type I and type IV collagen. The results revealed considerable variability in the collagenolytic potential of these bacteria, even among isolates of the same species. For *E. faecalis*, type I collagen activity ranged from 30 × 10^−3^ to 1320 × 10^−3^ relative fluorescence units (RFU)/s (mean 277 × 10^−3^), while for *P. aeruginosa* it ranged from 20 to 1916 RFU/s (mean 350 × 10^−3^). For *E. faecalis*, type IV collagen activity ranged from 7.6 × 10^−3^ to 920 × 10^−3^ RFU/s (mean 177 × 10^−3^), while for *P. aeruginosa* it ranged from 5.8 × 10^−3^ to 2086 × 10^−3^ RFU/s (mean 355 × 10^−3^).

Collagenase activity (as determined by the presence/absence of activity on skimmed milk plates) was detected in 52% of postoperative samples from patients with anastomotic leak (11 of 21) and 43% of postoperative samples from patients without anastomotic leak (33 of 77). This was not significant (Fisher’s exact test, *P* = 0.46). Neither type I (Mann–Whitney *U* test, *P* = 0.59) nor type IV (Mann–Whitney *U* test, *P* = 0.58) activity was different between postoperative samples from patients with and without anastomotic leak.

### Concordance between 16S rRNA sequencing and microbiological culture data

There was good concordance between culture results and 16S rRNA sequencing data (*Table 3*). As 16S reads were at the genus level, taxa were adjusted accordingly. Of the 14 collagenolytic genera identified, 8 showed significantly higher 16S read proportions in culture-positive samples. When comparing 16S read abundance across samples with collagenase activity (regardless of organisms), several genera were elevated, but only *Pseudomonas* remained significant after adjusting for multiple testing (*Table 3*).

## Discussion

This study provides valuable insights into the rectal microbiome of patients undergoing rectal cancer surgery, highlighting key factors associated with its composition and the changes that occur during the perioperative interval.

Patient-specific variability accounted for the largest proportion of beta-diversity, emphasizing the substantial individual differences in microbiome composition. These differences may be driven by a range of factors, including diet, environmental influences, host genetics, and previous microbial exposures^25^. The timing of sample collection was the second largest contributor to microbiome variation. As patients progressed through treatment, alpha-diversity decreased—typically a marker of a less healthy microbiome^26^. This likely reflects disruption from bowel preparation, surgery, and associated interventions. The reduction in alpha-diversity was accompanied by an increased abundance of taxa such as *Enterococcus* and *Prevotella* after surgery.

Smoking status was associated with variation in the baseline microbiome. Smokers exhibited distinct microbiome profiles, possibly due to smoking-induced changes such as elevated pH, low-grade inflammation, and oxidative stress^27^. In contrast, neoadjuvant therapy explained very little variation in beta-diversity at baseline. Mechanical bowel preparation was another relevant factor, particularly on the day of surgery. Differences were seen between rectal enema bowel preparation and oral mechanical bowel preparation, in contrast to previous findings by Zukauskaite *et al*.^28^ The use of preoperative oral antibiotics also showed associations with microbiome composition, both on the day of surgery and after surgery. However, only 8.4% of the study population received oral antibiotics, which may not accurately reflect current clinical practice, in light of growing evidence in favour of their use.^29–33^ The small number of patients who received oral antibiotics also limited the ability to further investigate microbiome differences based on the specific type of antibiotic used, an area that warrants further research.

Another observation was the variation in microbiome composition by hospital site on both the day of surgery and after surgery. The reason remains unclear, but could be related to differences in patient populations, local practices, or perioperative antibiotic prescribing protocols. Similar site-specific variation has been reported in other clinical settings^29^. The authors also observed that the presence of a defunctioning stoma was linked to a distinct postoperative microbiome profile, characterized by reduced alpha-diversity and an increased abundance of *Pseudomonas* and *Streptococcus*. The cause for this remains uncertain, though it is possible that the defunctioning stoma could serve as a surrogate for other unmeasured variables influencing the microbiome. The authors specifically considered this with respect to anastomotic height within their model, but this did not explain the observed differences, suggesting that this may be driven by factors not yet fully understood. Collectively, these results suggest that, despite strong individual variability, the microbiome appears responsive to several modifiable perioperative factors—offering potential avenues for clinical optimization.

Despite preclinical evidence linking the microbiome to anastomotic leak, no statistically significant difference in alpha- or beta-diversity was found between patients who developed anastomotic leak and those who did not. Although some differences in key taxa were observed, such as increases in *Hungatella* and *Eisenbergiella* intraoperatively and *Eubacterium* after surgery, as well as decreases in *Barnesiella* intraoperatively, these taxa were all present at relatively low levels, making the clinical significance of these data unclear.

A key aspect of the present study was the integration of culture-based analysis allowing collagenase-producing bacteria to be assessed. Preclinical studies have shown that collagenolytic enzymes play a crucial role in structural degradation of the anastomotic site. Guyton *et al*.^22^ demonstrated the utility of skimmed milk plates and a collagenase assay to isolate collagenolytic organisms and assess their collagenase production in four patients with anastomotic complications. To the authors’ knowledge, this is the first time such methods have been used in a larger clinical study. In the present study, *P. aeruginosa*, *E. faecalis*, and *P. mirabilis* were identified as the most commonly detected collagenase-producing organisms. These bacteria have been directly implicated in anastomotic leak in preclinical studies^7,30,31^. Additionally, the authors identified several less common bacterial species from clinical samples that exhibited collagenase activity, suggesting that this virulence factor may extend to other bacteria not previously associated with anastomotic leak.

Notably, the authors observed substantial variation in the collagenolytic potential of these organisms (as measured by activity against type I and type IV collagen), even among different isolates of the same species. This variability highlights the complexity of understanding the microbiome’s role in anastomotic leak and underscores the potential limitations of relying solely on sequencing data, which reflects the presence and relative abundance of bacteria, but does not capture important phenotypic characteristics.

Although collagenase activity was detected in a substantial proportion of postoperative samples (43.6%), no clear link with anastomotic leak was found. Nonetheless, the high prevalence of collagenase-producing organisms raises the possibility of preferential colonization at the anastomotic site in patients who develop anastomotic leak—a question that could not be addressed in the present study, as the anastomosis was not directly sampled. Preclinical studies suggest that the anastomotic environment can act as a chemoattractant for collagenase-producing organisms^32^. Further investigation in human subjects is warranted, though direct sampling poses practical and ethical challenges and will require careful planning. If site-specific colonization is confirmed, this could inform targeted antimicrobial or microbiome-modulating strategies aimed at reducing anastomotic leak.

Few studies have explored the potential relationship between the microbiome and clinical outcomes in colorectal surgery. Van Praagh *et al*. analysed the mucosal microbiome of anastomotic doughnuts from stapled colorectal anastomoses in 123 patients as part of the C-seal trial, finding that anastomotic leak was associated with low microbial diversity and a high abundance of Bacteroidaceae and Lachnospiraceae.^34^ Shogan *et al*. conducted a prospective study of 101 patients undergoing colorectal resections, collecting samples before surgery and on postoperative day 2, observing that patients who developed postoperative ileus had an increased abundance of *Bacteroides*, *Parabacteroides*, and *Ruminococcus*^35^. However, they found no significant microbiome differences in patients who developed surgical-site infections or anastomotic leaks.

The strengths of the present study include it being the largest study to date on the microbiome of rectal cancer patients undergoing surgery. It was conducted within a multicentre RCT with rigorous follow-up data, including the assessment of anastomotic leak via contrast radiology. The present study does have limitations. The data should be interpreted within the confines of the study population, which included a high proportion of white ethnicity and had an unexpectedly high circumferential resection margin positivity rate (19.8% *versus* <5% in the overall IntAct population). This may be partly due to a higher proportion of low anterior resections in the substudy. However, inter-site differences in imaging, staging, or operative technique may also have contributed. Another limitation is the fixed timing of microbiome sampling, which may have missed dynamic microbial changes in patients who developed anastomotic leak after the postoperative sampling window. Future studies should consider incorporating additional sampling time points closer to the time of anastomotic leak diagnosis to better capture the evolving microbiome in this setting.

In conclusion, patients undergoing rectal cancer surgery in this study demonstrated measurable microbiome changes during the perioperative interval. Factors such as smoking, bowel preparation, hospital site, and a defunctioning stoma had a notable impact on beta-diversity. Alpha-diversity decreased during treatment, with postoperative increases in *Enterococcus* and *Prevotella*. Although small differences in the microbiome were observed between patients with and without anastomotic leak, their clinical significance is unclear and requires further investigation. Importantly, the detection of collagenase-producing organisms—previously implicated in anastomotic leak in preclinical studies—merits further exploration, particularly regarding their potential for anastomotic colonization. These findings offer a foundation for future mechanistic and interventional studies aimed at optimizing the microbiome before surgery and should be validated in external cohorts.

## Supplementary Material

znaf199_Supplementary_Data

## Data Availability

Data sharing will be possible upon reasonable request for secondary research purposes. Requests to access trial data should be made to CTRU-DataAccess@leeds.ac.uk in the first instance. Requests will be reviewed by relevant stakeholders.
