# Supplementary material for: Perioperative changes in the microbiome during rectal cancer surgery: exploratory analysis of the National Institute for Health and Care Research (NIHR) IntAct trial
Source: Br J Surg. 2025 Sep 30;112(9):znaf199. doi: 10.1093/bjs/znaf199 (PMC12481238; doi:10.1093/bjs/znaf199)
Supplement: znaf199_Supplementary_Data [file znaf199_supplementary_data.docx]

**Perioperative changes in the microbiome during rectal cancer surgery: an exploratory analysis of the NIHR IntAct trial**

Jack A Helliwell^1^, Caroline H Chilton^1^, Caroline Young^2^, Emma V Clark^1^, Lyndsay Wilkinson^1^, Alba Fuentes Balaguer^1^, Daniel Bottomley^1^, Julie Croft^3^, Neil Corrigan^3^, Andrew Kirby^1^, Philip Quirke^1^, Deborah D Stocken^3^, David G Jayne^1^, Henry M Wood^1^

1. Leeds Institute of Medical Research at St. James’s, University of Leeds, Leeds, UK
2. Leeds Teaching Hospitals NHS Trust, Leeds, UK
3. Leeds Institute of Clinical Trials Research, University of Leeds, Leeds, UK

**Address Correspondence to:** Jack A Helliwell, Leeds Institute of Medical Research at St. James’s, University of Leeds, LS9 7TF. Email: [j.a.helliwell@leeds.ac.uk](mailto:j.a.helliwell@leeds.ac.uk)

**Supplementary Materials - Index**

| **Supplementary Results** |  |
| --- | --- |
| Supplementary Table 1. Results of MaAsLin2 multivariate comparison of bacterial taxa to metadata categories. Only taxa with significant associations are shown. For each, the genus, metadata category, the metadata value, coefficient (positive indicates gain of that genus in with that value), standard error, sample numbers, p-value and q-value are shown. | *page 2* |
|  |  |

| **Feature** | **metadata** | **value** | **coef** | **stderr** | **N** | **N.not.0** | **pval** | **qval** |
| --- | --- | --- | --- | --- | --- | --- | --- | --- |
| Enterococcus | Timepoint | Post-op | 4.66045305 | 0.77912432 | 310 | 245 | 7.84E-09 | 3.04E-05 |
| Lachnospiraceae.NK4A136.group | Timepoint | Post-op | -3.6709144 | 0.66802474 | 310 | 232 | 1.04E-07 | 0.00017304 |
| Lachnospiraceae.UCG.004 | Timepoint | Post-op | -3.5394265 | 0.65064923 | 310 | 188 | 1.34E-07 | 0.00017304 |
| Odoribacter | Defunctioning stoma | Yes | -3.0460314 | 0.60361125 | 310 | 287 | 8.85E-07 | 0.00085772 |
| Fusobacterium | Timepoint | Post-op | -3.9575565 | 0.79078699 | 310 | 247 | 1.22E-06 | 0.00094802 |
| Bacteroides | Defunctioning stoma | Yes | -1.8684609 | 0.40189177 | 310 | 310 | 5.24E-06 | 0.00309227 |
| Dorea | Timepoint | Post-op | -2.5799945 | 0.55523596 | 310 | 275 | 5.59E-06 | 0.00309227 |
| Lachnoclostridium | Defunctioning stoma | Yes | -3.038334 | 0.67068467 | 310 | 270 | 8.78E-06 | 0.00378101 |
| Morganella | Ethnicity | Asian - Pakistani | 7.4267981 | 1.61315515 | 310 | 48 | 8.70E-06 | 0.00378101 |
| Parabacteroides | Defunctioning stoma | Yes | -2.1259347 | 0.4977014 | 310 | 310 | 2.70E-05 | 0.00949871 |
| Prevotella | Timepoint | Post-op | 4.54367544 | 1.05768291 | 310 | 203 | 2.58E-05 | 0.00949871 |
| Comamonas | Ethnicity | Asian - Indian | 10.4096044 | 2.42897206 | 310 | 95 | 3.51E-05 | 0.01133037 |
| Anaerostipes | Timepoint | Post-op | -2.4129619 | 0.58433903 | 310 | 295 | 4.91E-05 | 0.01463102 |
| Finegoldia | Timepoint | Post-op | 3.64401637 | 0.89445816 | 310 | 223 | 6.18E-05 | 0.0171107 |
| Faecalibacterium | Timepoint | Post-op | -1.7537182 | 0.43565903 | 310 | 309 | 7.79E-05 | 0.02012439 |
| Roseburia | Timepoint | Post-op | -2.374517 | 0.59952857 | 310 | 253 | 9.98E-05 | 0.02417719 |
| Prevotella.2 | Ethnicity | Other Asian | 5.56151236 | 1.44406486 | 310 | 46 | 0.00015815 | 0.03604974 |
| Lactobacillus | Type of bowel prep | None | 9.76456465 | 2.60520518 | 310 | 149 | 0.00024656 | 0.05267305 |
| Lachnospiraceae.FCS020.group | Timepoint | Post-op | -2.1711659 | 0.58541313 | 310 | 109 | 0.00025827 | 0.05267305 |
| Eikenella | Timepoint | Post-op | 2.27322977 | 0.6171379 | 310 | 63 | 0.00028819 | 0.05583604 |
| Eubacterium | Defunctioning stoma | Yes | -1.4151838 | 0.38586948 | 310 | 47 | 0.0003112 | 0.05742416 |
| Coprococcus.1 | Timepoint | Post-op | -2.1076814 | 0.58207873 | 310 | 271 | 0.00036399 | 0.0641122 |
| Lawsonella | Defunctioning stoma | Yes | 2.40075098 | 0.68038647 | 310 | 71 | 0.00049087 | 0.06660127 |
| Barnesiella | Timepoint | Post-op | -1.9130632 | 0.54120669 | 310 | 300 | 0.00049734 | 0.06660127 |
| Lactobacillus | Timepoint | baseline | -8.9438473 | 2.48566385 | 310 | 149 | 0.00043119 | 0.06660127 |
| Clostridium.sensu.stricto.1 | Age | Age | 0.48162008 | 0.13551132 | 310 | 295 | 0.00051735 | 0.06660127 |
| Peptoniphilus | Timepoint | Post-op | 3.05771945 | 0.8641481 | 310 | 226 | 0.0004796 | 0.06660127 |
| Coprococcus.3 | Timepoint | Post-op | -2.2363611 | 0.63496957 | 310 | 231 | 0.00052058 | 0.06660127 |
| Hungatella | Anastomotic leak | Yes | 2.18245672 | 0.60777643 | 310 | 153 | 0.00044801 | 0.06660127 |
| Lachnospira | Timepoint | Post-op | -2.5862015 | 0.73607852 | 310 | 207 | 0.00053281 | 0.06660127 |
| Sellimonas | Defunctioning stoma | Yes | -1.78361 | 0.4997035 | 310 | 53 | 0.00044576 | 0.06660127 |
| Ruminococcaceae.UCG.014 | Sex | Female | -1.5406148 | 0.44501196 | 310 | 263 | 0.00073285 | 0.08874336 |
| Eubacterium | Timepoint | Post-op | 1.14333283 | 0.33720354 | 310 | 47 | 0.00084453 | 0.09625106 |
| [Ruminococcus].gnavus.group | Tumour position | At peritoneal reflection | -2.0131509 | 0.58774502 | 310 | 206 | 0.00084321 | 0.09625106 |
| Erysipelotrichaceae.UCG.003 | Timepoint | Post-op | -1.5514541 | 0.46032647 | 310 | 288 | 0.00089001 | 0.09853681 |
| Ruminiclostridium.9 | Ethnicity | Asian - Indian | -5.4890568 | 1.62250695 | 310 | 240 | 0.0009169 | 0.09869406 |
| Sellimonas | Timepoint | Post-op | 1.45184658 | 0.43625747 | 310 | 53 | 0.00104798 | 0.10188047 |
| Flavonifractor | Defunctioning stoma | Yes | -2.4502853 | 0.73439499 | 310 | 239 | 0.0009748 | 0.10188047 |
| Ruminococcaceae.UCG.003 | Timepoint | Post-op | -2.0914739 | 0.62681994 | 310 | 219 | 0.00100039 | 0.10188047 |
| Ruminococcaceae.UCG.005 | Timepoint | Post-op | -2.0874943 | 0.62932874 | 310 | 276 | 0.00105167 | 0.10188047 |
| Ruminiclostridium.5 | Timepoint | Post-op | -1.8623133 | 0.564347 | 310 | 261 | 0.00111421 | 0.10530607 |
| Coprococcus.1 | Defunctioning stoma | Yes | -2.1649903 | 0.65946674 | 310 | 271 | 0.00117942 | 0.10628486 |
| Ruminococcaceae.NK4A214.group | Sex | Female | -1.5372767 | 0.46312314 | 310 | 240 | 0.00116521 | 0.10628486 |
| Ruminococcus.2 | Type of bowel prep | Mechanical_Enema | -2.3368333 | 0.71690255 | 310 | 310 | 0.00125259 | 0.1103129 |
| Coprobacter | Smoking | Never smoker | -3.3541706 | 1.02201205 | 310 | 182 | 0.00132617 | 0.11419811 |
| Peptoniphilus | Age | Age | -0.7921339 | 0.24256065 | 310 | 226 | 0.00143541 | 0.12043327 |
| Cloacibacillus | Age | Age | 0.52496235 | 0.16107992 | 310 | 37 | 0.00146074 | 0.12043327 |
| Catenibacterium | Tumour position | Below peritoneal reflection | -1.3835545 | 0.42811642 | 310 | 110 | 0.00150324 | 0.12135556 |
| Butyricimonas | Defunctioning stoma | Yes | -2.5046679 | 0.78232015 | 310 | 223 | 0.00157098 | 0.1242354 |
| Corynebacterium.1 | Age | Age | -0.6813135 | 0.21147974 | 310 | 89 | 0.00161401 | 0.12508582 |
| Coprobacter | Smoking | Ex smoker | -3.3667122 | 1.0635179 | 310 | 182 | 0.00192453 | 0.14622666 |
| Haemophilus | Ethnicity | Black Caribbean | 7.90923243 | 2.49786107 | 310 | 262 | 0.00197343 | 0.1470584 |
| Ruminococcaceae.UCG.004 | Age | Age | -0.831492 | 0.26861837 | 310 | 165 | 0.00237371 | 0.17354953 |
| Prevotella.6 | Sex | Female | -1.5354899 | 0.49467448 | 310 | 111 | 0.00242829 | 0.17425213 |
| Gemella | Neo-adjuvant therapy | Yes | -2.8047355 | 0.91177645 | 310 | 176 | 0.0025159 | 0.17725624 |
| Coprococcus.1 | Ethnicity | Asian - Indian | -4.7407101 | 1.57024181 | 310 | 271 | 0.0030707 | 0.20050001 |
| Anaerotruncus | Ethnicity | Asian - Pakistani | -7.4614473 | 2.46499941 | 310 | 230 | 0.00296823 | 0.20050001 |
| Ruminiclostridium.9 | Defunctioning stoma | Yes | -2.4352469 | 0.81636439 | 310 | 240 | 0.00310452 | 0.20050001 |
| Parasutterella | Neo-adjuvant therapy | Yes | -1.8583171 | 0.61521878 | 310 | 266 | 0.00300182 | 0.20050001 |
| Akkermansia | Ethnicity | Not given | 8.70152359 | 2.90895955 | 310 | 308 | 0.00308479 | 0.20050001 |
| Mobiluncus | Age | Age | -0.430392 | 0.14569241 | 310 | 57 | 0.00339817 | 0.20107736 |
| Blautia | Ethnicity | Asian - Pakistani | -3.7188738 | 1.24107112 | 310 | 309 | 0.00328201 | 0.20107736 |
| Ruminococcaceae.UCG.002 | Timepoint | Post-op | -1.2983157 | 0.43627874 | 310 | 309 | 0.00326026 | 0.20107736 |
| Ruminococcus.2 | Type of bowel prep | Mechanical | -1.8649244 | 0.6318172 | 310 | 310 | 0.0034248 | 0.20107736 |
| Morganella | Ethnicity | Not given | 6.18473108 | 2.08015108 | 310 | 48 | 0.00322389 | 0.20107736 |
| Haemophilus | Age | Age | 0.61672783 | 0.20581872 | 310 | 262 | 0.00334375 | 0.20107736 |
| Akkermansia | Defunctioning stoma | Yes | -1.8936721 | 0.6430297 | 310 | 308 | 0.00357448 | 0.2067332 |
| [Ruminococcus].torques.group | Ethnicity | Asian - Indian | -4.0079358 | 1.36063936 | 310 | 289 | 0.00372325 | 0.21217055 |
| Haemophilus | T-stage | T4a | -6.0233818 | 2.04690183 | 310 | 262 | 0.00386666 | 0.21714965 |
| Ezakiella | Age | Age | -0.6282823 | 0.21389141 | 310 | 116 | 0.00396116 | 0.21927868 |
| Collinsella | Defunctioning stoma | Yes | -1.5865878 | 0.54666143 | 310 | 285 | 0.00404681 | 0.21974255 |
| Anaerococcus | Age | Age | -0.802126 | 0.27513055 | 310 | 212 | 0.00417198 | 0.21974255 |
| Gallicola | Defunctioning stoma | Yes | 1.24216776 | 0.43182046 | 310 | 33 | 0.00435225 | 0.21974255 |
| Ruminiclostridium.9 | Ethnicity | Asian - Pakistani | -6.7472238 | 2.32086838 | 310 | 240 | 0.00420877 | 0.21974255 |
| [Eubacterium].coprostanoligenes.group | T-stage | T2 | 3.79420008 | 1.30453529 | 310 | 294 | 0.00432689 | 0.21974255 |
| Megamonas | Oral antibiotics | Yes | 2.69129735 | 0.93393443 | 310 | 83 | 0.00433774 | 0.21974255 |
| Oxalobacter | Ethnicity | Not given | 3.86685838 | 1.34516472 | 310 | 33 | 0.0043665 | 0.21974255 |
| [Eubacterium].eligens.group | Type of bowel prep | Mechanical | -2.6736737 | 0.9405749 | 310 | 197 | 0.0048 | 0.23544302 |
| Desulfovibrio | Sex | Female | -1.8957712 | 0.6609679 | 310 | 231 | 0.00479251 | 0.23544302 |
| Eisenbergiella | Anastomotic leak | Yes | 1.40153641 | 0.49108171 | 310 | 121 | 0.00495465 | 0.23726974 |
| [Eubacterium].ventriosum.group | Timepoint | Post-op | -1.7744903 | 0.62651875 | 310 | 151 | 0.00500358 | 0.23726974 |
| Ruminiclostridium.5 | Defunctioning stoma | Yes | -1.8005788 | 0.63627687 | 310 | 261 | 0.00502093 | 0.23726974 |
| Family.XIII.UCG.001 | Sex | Female | -1.1548561 | 0.40521246 | 310 | 125 | 0.00516128 | 0.23809485 |
| Erysipelotrichaceae.UCG.004 | Ethnicity | Not given | 5.08565132 | 1.8000422 | 310 | 35 | 0.0051527 | 0.23809485 |
| Veillonella | Ethnicity | Black Caribbean | 6.74668558 | 2.37719431 | 310 | 132 | 0.00522577 | 0.23823376 |
| Coprobacter | Sex | Female | -1.7600293 | 0.62522708 | 310 | 182 | 0.00562295 | 0.24269006 |
| Christensenellaceae.R.7.group | Timepoint | Post-op | -1.1508478 | 0.41095986 | 310 | 309 | 0.00563667 | 0.24269006 |
| Lachnoclostridium | Ethnicity | Asian - Indian | -3.9021021 | 1.38687749 | 310 | 270 | 0.0055619 | 0.24269006 |
| Ruminococcus.2 | Sex | Female | -0.8905434 | 0.31495789 | 310 | 310 | 0.0054833 | 0.24269006 |
| Bilophila | Timepoint | baseline | 5.59876667 | 1.98067923 | 310 | 301 | 0.0054134 | 0.24269006 |
| Succinivibrio | Smoking | Ex smoker | -1.5070265 | 0.53696065 | 310 | 270 | 0.00577222 | 0.2457951 |
| [Eubacterium].eligens.group | Timepoint | Post-op | -1.8197163 | 0.65531826 | 310 | 197 | 0.00596157 | 0.24839895 |
| Phascolarctobacterium | Sex | Female | -1.7225443 | 0.61605757 | 310 | 207 | 0.00593301 | 0.24839895 |
| Arcanobacterium | Timepoint | Post-op | 1.27090912 | 0.45897979 | 310 | 37 | 0.0060267 | 0.24844091 |
